# Inhibition of PRMT1 alleviates sepsis‐induced acute kidney injury in mice by blocking the TGF‐β1 and IL‐6 trans‐signaling pathways

**DOI:** 10.1002/2211-5463.13684

**Published:** 2023-08-12

**Authors:** Yu Zhu, Longmei Wang, Rui Liu, Xiurong Ding, Song Yin, Yuankun Chen, Chuanlong Zhu, Zheng Wang, Wenting Li

**Affiliations:** ^1^ Nephrology Department, Shenzhen Hospital University of Chinese Academy of Sciences (Guangming) Shenzhen China; ^2^ Department of Infectious Diseases Enze Medical Center Linhai China; ^3^ Department of Infectious and Tropical Diseases The Second Affiliated Hospital of Hainan Medical University Haikou China; ^4^ National Health Commission Key Laboratory of Tropical Disease Control Hainan Medical University Haikou China; ^5^ Graduate School of Bengbu Medical College China; ^6^ Division of Life Sciences and Medicine, Department of Infectious Disease, The First Affiliated Hospital of USTC University of Science and Technology of China Hefei China; ^7^ Wannan Medical College Wuhu China; ^8^ Department of Infectious Disease The First Affiliated Hospital of Nanjing Medical University China; ^9^ Department of Respiratory and Critical Medicine People's Hospital of Zhengzhou University China; ^10^ Department of Infectious Disease The First Affiliated Hospital of Anhui Medical University Hefei China

**Keywords:** acute kidney injury, IL‐6, protein arginine methyltransferase‐1, sepsis, TGF‐β1

## Abstract

Sepsis‐induced acute kidney injury (SI‐AKI) causes renal dysfunction and has a high mortality rate. Protein arginine methyltransferase‐1 (PRMT1) is a key regulator of renal insufficiency. In the present study, we explored the potential involvement of PRMT1 in SI‐AKI. A murine model of SI‐AKI was induced by cecal ligation and perforation. The expression and localization of PRMT1 and molecules involved in the transforming growth factor (TGF)‐β1/Smad3 and interleukin (IL)‐6/signal transducer and activator of transcription 3 (STAT3) signaling pathways were detected in mouse kidney tissues by western blot analysis, immunofluorescence, and immunohistochemistry. The association of PRMT1 with downstream molecules of the TGF‐β1/Smad3 and IL‐6/STAT3 signaling pathways was further verified *in vitro* in mouse renal tubular epithelial cells. Cecal ligation and perforation caused epithelial–mesenchymal transition, apoptosis, and inflammation in renal tissues, and this was alleviated by inhibition of PRMT1. Inhibition of PRMT1 in SI‐AKI mice decreased the expression of TGF‐β1 and phosphorylation of Smad3 in the renal cortex, and downregulated the expression of soluble IL‐6R and phosphorylation of STAT3 in the medulla. Knockdown of PRMT1 in mouse renal tubular epithelial cells restricted the expression of Cox‐2, E‐cadherin, Pro‐caspase3, and phosphorylated Smad3 (involved in the TGF‐β1‐mediated signaling pathway), and also blocked IL‐6/soluble IL‐6R, inducing the expression of Cox‐2 and phosphorylated‐STAT3. In conclusion, our findings suggest that inhibition of PRMT1 mitigates SI‐AKI by inactivating the TGF‐β1/Smad3 pathway in the cortex and the IL‐6/STAT3 pathway in the medulla. Our findings may aid in the identification of potential therapeutic target molecules for SI‐AKI.

## Graphical abstract

Protein arginine methyltransferase‐1 (PRMT1) is a key regulator of renal dysfunction caused by sepsis‐induced acute kidney injury (SI‐AKI). Inhibition of PRMT1 alleviates SI‐AKI by inactivating the transforming growth factor (TGF)‐β1/Smad3 pathway in the cortex and interleukin (IL)‐6/signal transducer and activator of transcription 3 (STAT3) pathway in the medulla. PRMT1 may be a potential therapeutic target molecule for SI‐AKI.

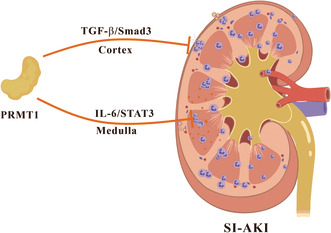

**Abbreviations**
AMI‐1: arginine methyltransferase inhibitor 1
ANOVA: analysis of variance
BUN: blood urine nitrogen
CLP: cecal ligation and perforation
Cr: creatinine
EMT: epithelial–mesenchymal transition
IL: interleukin
JAK/STAT3: Janus kinase/signal transducer and activator of transcription
MMP: matrix metalloproteinase
mRTEC: mouse renal tubular epithelial cell
NGAL: neutrophil gelatinase‐associated lipocalin
PRMT1: protein arginine methyltransferase‐1
p‐Smad3: phosphorylated Smad3
pSTAT: phosphorylated STAT3
SI‐AKI: sepsis‐induced acute kidney injury
sIL‐6R: soluble IL‐6R
si‐NC: siRNA control
si‐PRMT1: siRNA targeting PRMT1
siRNA: small interfering RNA
TGF: transforming growth factor
TIMP: tissue inhibitor of metalloproteinase
TUNEL: terminal deoxynucleotidyl transferase dUTP nick end labeling

Sepsis is a systemic inflammatory process in response to infections [1, 2] and an independent risk factor for in‐hospital deaths [3, 4]. It is the most common cause of acute kidney injury (AKI) in intensive care unit patients, and the incidence of renal damage during sepsis is as high as 48.1% [5]. However, AKI in sepsis is associated with a poor prognosis [6, 7], and the mortality rate of sepsis patients with AKI is three‐ to five‐fold higher than that of sepsis patients without AKI [8]. Thus, preventing sepsis from causing AKI remains an important topic.

Protein arginine methylation, a post‐translational modification, is involved in multiple physiological processes, such as cell cycle control, immune responses, apoptosis, and oxidative stress responses [9]. The Protein arginine methyltransferase‐1 (PRMT1) is a predominant enzyme responsible for cellular arginine methylation [10], and plays a pro‐inflammatory role in pulmonary fibrosis by activating the extracellular signal regulated kinase pathway [11]. Additionally, apoptosis of podocytes and epithelial–mesenchymal transition (EMT) during diabetic nephropathy were suppressed by arginine methyltransferase inhibitor 1 (AMI‐1), a selective inhibitor for PRMT1 [12]. Currently, only a few studies have explored the role and mechanism of PRMT1 in AKI.

Transforming growth factor (TFG)‐β1, the most abundant isoform of TGF‐β family members, can be released by all types of renal cells and infiltrating inflammatory cells [13]. TGF‐β1 is secreted from injured tubular epithelial cells and results in the activation of TGF‐β1 signaling in adjacent renal fibroblasts upon damage to the kidneys [14]. TGF‐β1/Smad3 signaling induced by miR‐207 promotes renal tissue fibrosis [15]. PRMT1 can promote renal fibrosis injured by left ureteral obstruction through the TGF‐β1/Smad3 signaling pathway [16], suggesting a key role of PRMT1 in TGF‐β1/Smad3 signaling.

Current studies suggest that sepsis may initiate a cytokine storm, where non‐specific immune cells, mainly macrophages, release various cytokines upon activation [17]. Once sepsis initiates, interleukin (IL)‐6 is rapidly released and reaches the peak level within a considerable short amount of time [18]; the severity of which corresponds to that of infection [19]. IL‐6R, the membrane receptor of IL‐6, could also be differentially spliced and translated to produce soluble IL‐6R (sIL‐6R) in the cytoplasm. Hence, IL‐6R and sIL‐6R provide different signaling for IL‐6: classic and trans‐signaling pathways [20, 21]. In the classic signaling pathway, IL‐6 binds to IL‐6R in the cell membrane and connects with gp130, which initiates the Janus kinase/signal transducer and activator of transcription (JAK/STAT3) pathway [22]. In the trans‐signaling pathway, sIL‐6R binds to IL‐6 with a comparable affinity to that of IL‐6R. The sIL‐6R/IL‐6 complex then initiates gp130 and results in the activation of JAK/STAT3 pathway [23, 24]. Both the classic and trans‐signaling pathways lead to the phosphorylation of STAT3 [20, 25]. A previous study reports that IL‐6 trans‐signaling pathway plays an important role in the initiation and progression of renal fibrosis [26]. Additionally, TGF‐β1 initiates the IL‐6/STAT3 trans‐signaling pathway [27], which can be augmented by IL‐6 in a positive feedback manner [28]. PRMT1 is shown to methylate the arginine residues on STAT3 protein, a downstream effector of IL‐6 trans‐signaling pathways, thereby positively regulating its activity [29]; however, the direct regulation of PRMT1 to IL‐6 trans‐signaling pathway is unknown.

Based on these studies, PRMT is a key regulator in the TGF‐β1/Smad3 signaling pathway and STAT3 signaling and functions in renal fibrosis. Thus, in the present study, we utilized the cecal ligation and perforation (CLP) murine model, a classic model of SI‐AKI, to assess whether the progression of sepsis‐induced renal injury is epigenetically regulated by PRMT1 through the TGF‐β1/Smad3 and IL‐6/STAT3 pathways. Our findings may provide new insight into the pathogenesis of SI‐AKI, and a new therapeutic target for the SI‐AKI treatment.

## Results

**Fig. 1:**
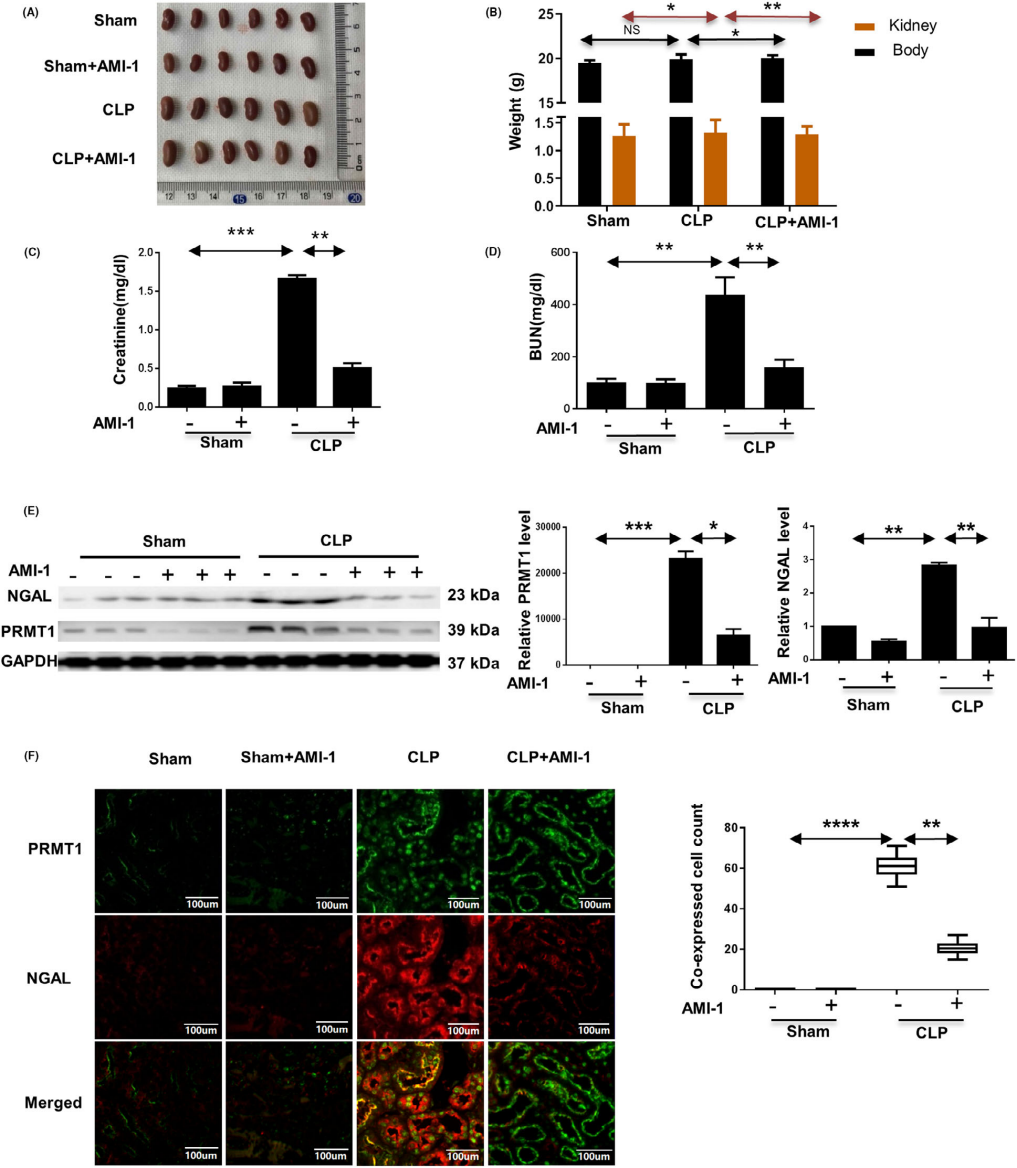
Inhibition of PRMT1 mediated by AMI‐1 mitigates CLP‐induced murine kidney injury. (A) Images of mice kidneys collected from the right abdominal cavity. (B) Weight of mice and right kidneys from the sham, CLP, and CLP with AMI‐1 groups. (C, D) The serum levels of Cr (C) and BUN (D) of mice from each group. (E) Representative images of the western blot assay and relative protein expression levels of NGAL and PRMT1 in the kidneys. (F) Double immunofluorescence staining of PRMT1 (green) and NGAL (red), and the number of cells co‐expressing PRMT1 and NGAL (yellow) in kidney. Data are expressed as the mean ± SD and analyzed using one‐way ANOVA with Tukey's post‐hoc test. All data were obtained from three independent experiments. *P < 0.05, **P < 0.01, ***P < 0.001, and ****P < 0.0001. Scale bar = 100 μm. AMI‐1, arginine methyltransferase inhibitor 1; BUN, blood urine nitrogen; Cr, Creatinine; CLP, cecal ligation and perforation; PRMT1, protein arginine methyltransferase‐1.

To explore the relationship between the expression of PRMT1 gene and SI‐AKI, AMI‐1, which acts as an inhibitor of PRMT1, was selected to reverse verify their relationship. All mice and their right kidneys were weighed 18 h after operations (Fig. 1A,B). Compared with the sham group, the whole‐body weight of the mice in the CLP group did not change significantly; however, the weight of their right kidney increased (*P* < 0.05). Conversely, the whole‐body and reduced kidney weights of mice in the CLP group with AMI‐1 treatment were lighter than those of CLP mice (*P* < 0.05 and *P* < 0.01, respectively). Moreover, the blood biochemistry assays showed that the CLP operation group had an increased creatinine (Cr) and blood urine nitrogen (BUN) in the serum, whereas that of the AMI‐1 treatment group decreased as a result of the increased CLP (*P* < 0.01) (Fig. 1C,D). The results showed that inhibition of PRMT1 expression alleviated AKI in septic patients. To further verify that PRMT1 gene expression is associated with SI‐AKI, we detected the expression of neutrophil gelatinase‐associated lipocalin (NGAL), a marker protein of AKI in different groups of mice using a western blot assay and immunofluorescence double staining. In the western blot analysis (Fig. 1E), PRMT1 was not expressed in the kidneys of the mice from the sham and the sham with AMI‐1 groups; conversely, it was drastically enhanced as a result of CLP operation and subsequently reduced following AMI‐1 treatment (*P* < 0.05). Meanwhile, the expression of NGAL in the CLP group was significantly higher than that in the sham group (*P* < 0.01) and AMI‐1 treatment caused the decreased expression of NGAL (*P* < 0.01). In immunofluorescence double staining, the expression of PRMT1 and NGAL was rarely visualized in the sham and sham with AMI‐1treatment groups but abundant in CLP‐operated mice (Fig. 1F). Notably, AMI‐1 inhibited the expression of PRMT1 in the CLP group. Additionally, in the CLP group, PRMT1 (nucleus) and NGAL (cytoplasm) expressed more highly in the same renal tubular epithelial cells; however, their respective expression and co‐expression were significantly reduced (*P* < 0.01) after AMI‐1 treatment. Therefore, PRMT1, similar to NGAL, is a marker of SI‐AKI.

**Fig. 2:**
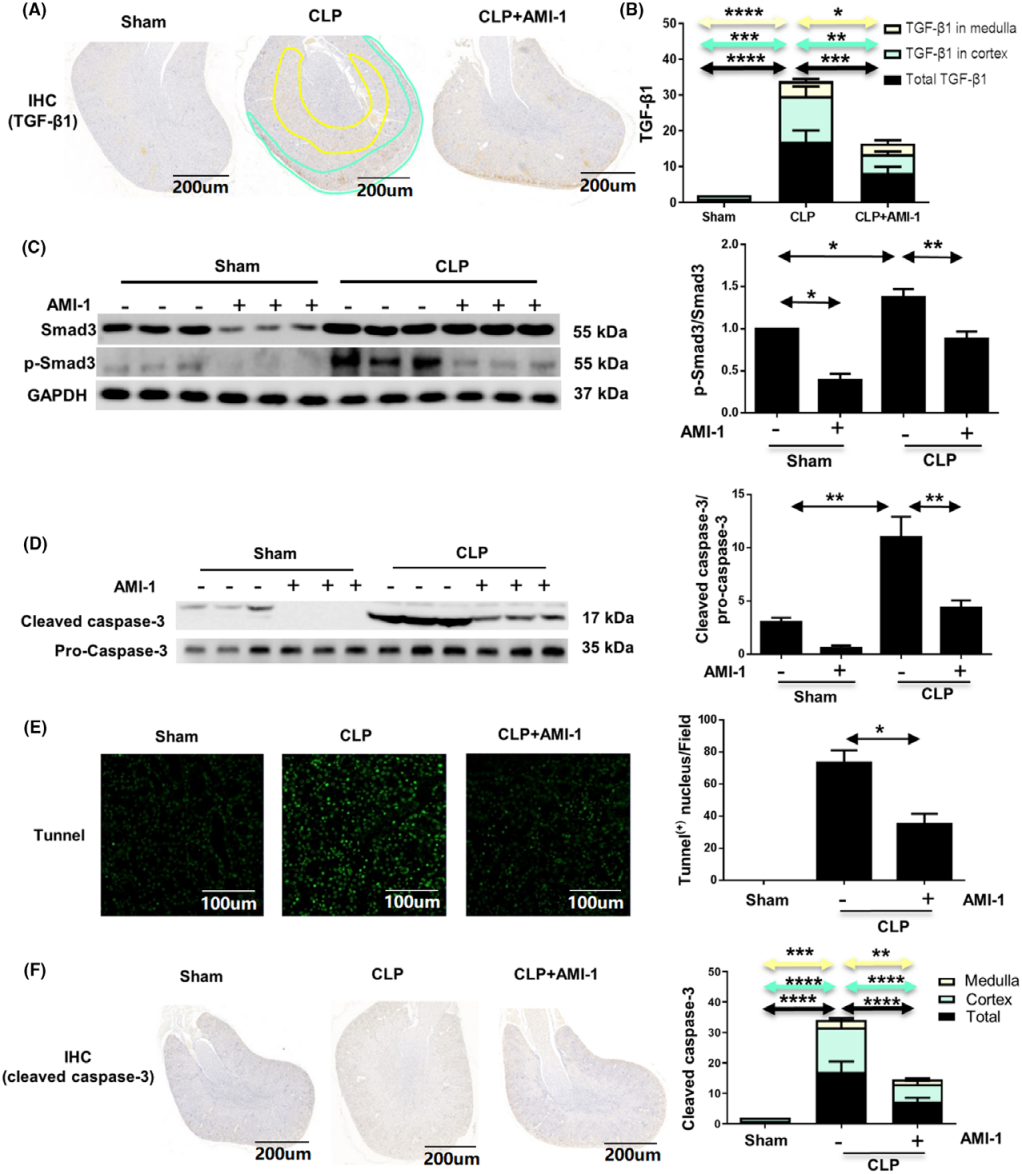
Inhibition of PRMT1 blocks the expression of TGF‐β1 and p‐Smad3, apoptosis in SI‐AKI. (A) Immunohistochemistry staining of TGF‐β1 on mouse kidneys from the sham, CLP, and CLP with AMI‐1 groups. Scale bar = 200 μm. (B) Quantitative analysis of TGF‐β1 expression in the stained area (medulla, cortex, and whole kidney) in each group. The positive staining for TGF‐β1 was detected in 10 high‐power fields. (C) Western blot images of total Smad3 and p‐Smad3 in the kidney tissues and relative protein expression levels of p‐Smad3 to total Smad3. (D) Western blot assay of cleaved caspase‐3 and pro‐cleaved caspase‐3 and the relative protein expression levels of cleaved caspase‐3 to pro‐cleaved caspase‐3 in the kidneys. (E) TUNEL staining and the number of TUNEL‐positive cells in mouse kidneys from the sham, CLP, and CLP with AMI‐1 groups. Scale bar = 100 μm. (F) Immunohistochemical staining of cleaved caspase‐3 and quantitative analysis of cleaved caspase‐3 in the stained area (medulla, cortex, or the whole kidney) in each group. Scale bar = 200 μm. Data are expressed as the mean ± SD and analyzed using one‐way ANOVA with Tukey's post‐hoc test. All data were obtained from three independent experiments. *P < 0.05, **P < 0.01, and ***P < 0.001 and ****P < 0.0001. AMI‐1, arginine methyltransferase inhibitor 1; CLP, cecal ligation and perforation; PRMT1, protein arginine methyltransferase‐1; SI‐AKI, Sepsis‐induced acute kidney injury.

TGF‐β1 is the most common initiating factor among various acute and chronic injury factors of renal tubular epithelial cells in SI‐AKI [13], and our results showed that PRMT1 was marker of SI‐AKI. Thus, to detect whether there is an association between PRMT1 and TGF‐β1 in SI‐AKI, we performed immunohistochemical staining of TGF‐β1 in renal pathological sections. TGF‐β1 was highly expressed in the cortical renal tubular epithelial cells from the mouse kidney in the CLP group (Fig. 2A, blue circle), some of which were also detected in the interstitial region of the tubular epithelium (Fig. 2A, yellow circle). The quantification of immunohistochemical staining showed that the overall expression of TGF‐β1 in the medullary region was remarkably less than that in the cortex. CLP induced the upregulation of TGF‐β1 expression in the medulla, cortex, and whole kidney, whereas AMI‐1 markedly inhibited its expression in these regions (Fig. 2B). Smad3 protein, downstream of TGF‐β1 signal pathway, was quantitatively in the whole kidney using western blot analysis (Fig. 2C). The expression of phosphorylated Smad3 was significantly enriched in mice from the CLP group. However, the level of total Smad3 protein was not significantly different from that in the sham group. The relative increasing expression of phosphorylated Smad3 to total Smad3 indicates that Smad3 was significantly activated in the kidney after CLP (*P* < 0.05) and the extent of activation was weakened by AMI‐1 treatment (*P* < 0.01). Smad3 phosphorylation promotes the repair of inflammatory cells, but results in epithelial phenotype transition [15, 16]. Hence, in the renal cortex, we consider that PRMT1 regulates the epithelial phenotype transition through TGF‐β1 signal pathway in SI‐AKI.

The activated TGF‐β1 signal pathway has many biological functions. To explore the relationship between PRMT1 and apoptosis in SI‐AKI, we detected several parameters related to the extent and region of apoptosis in mice in each group. The results of the western blot showed that sepsis induced by CLP increased the expression of cleaved caspase‐3 in the renal tissues, and AMI repressed the cleavage of caspase‐3 (*P* < 0.01) (Fig. 2D), suggesting that AMI might reduce cell apoptosis by inhibiting PRMT1 molecule in the kidneys. Meanwhile, the results of terminal deoxynucleotidyl transferase dUTP nick end labeling (TUNEL) staining showed that apoptosis mainly occurred in the renal tubular epithelial cells damaged by sepsis, and AMI‐1 reduced the number of TUNEL‐positive cells (*P* < 0.05) (Fig. 2E). The expression of cleaved caspase‐3 in CLP group was significantly higher than that in the sham group, which concentrated mostly in the cortical tubules and slightly in the medulla. AMI‐1 addition reduced the expression of cleaved caspase‐3 (*P* < 0.01) (Fig. 2F). This suggests that PRMT1 inhibition alleviates sepsis‐induced apoptosis in the kidneys, particularly in renal tubular epithelial cells.

**Fig. 3:**
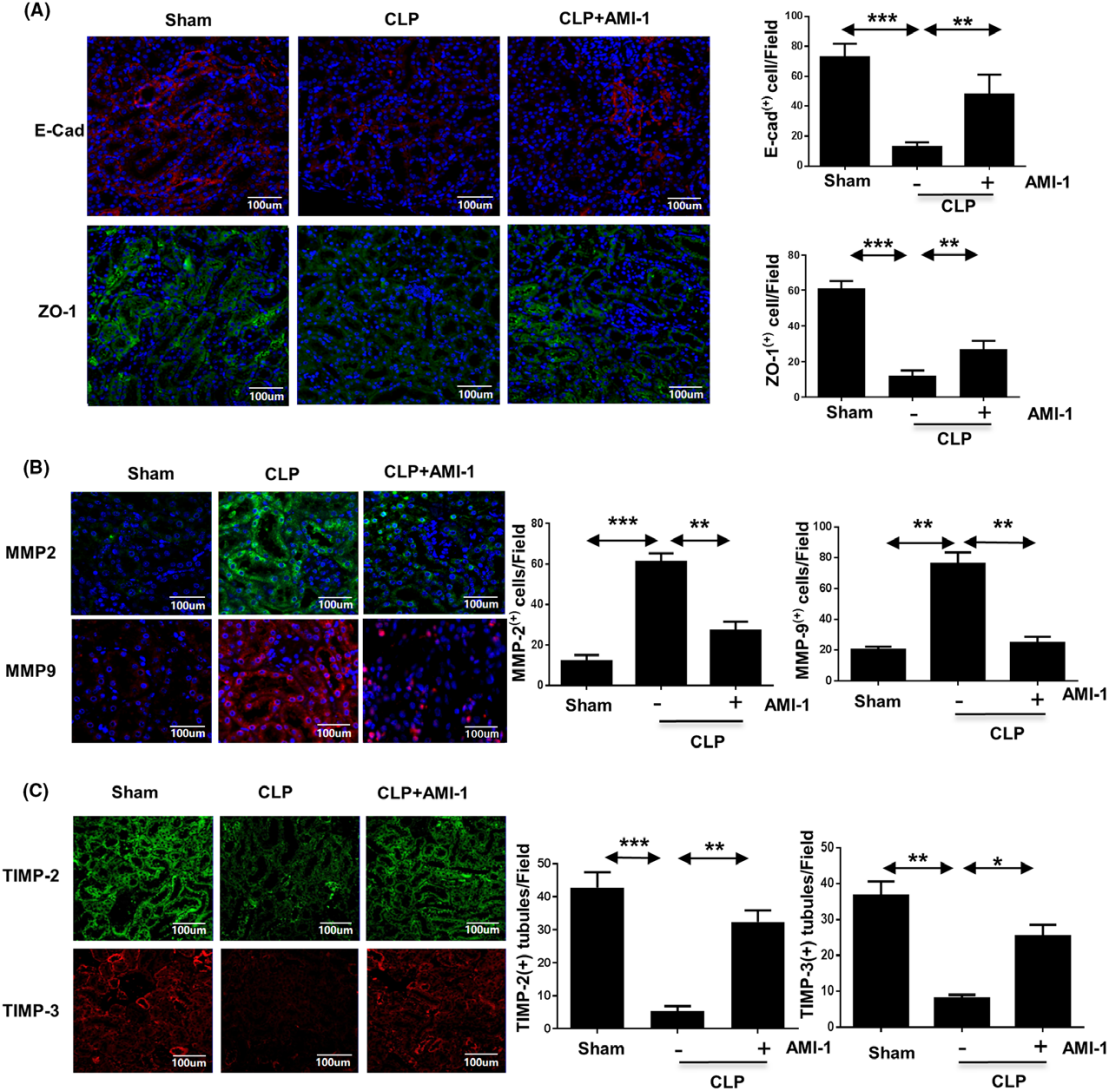
Inhibition of PRMT1 suppresses EMT in SI‐AKI. (A) Immunofluorescence staining of E‐cadherin and ZO‐1 in murine kidney tissues and the numbers of E‐cadherin^(+)^ and ZO‐1^(+)^ cells in each field. (B) Immunofluorescence staining of MMP‐9 and MMP‐2 in murine kidney tissues and the number of MMP‐9^(+)^ and MMP‐2^(+)^ cells in each field. (C) Immunofluorescence staining of TIMP‐2 and TIMP‐3 and the number of double‐positive cells). Tubular cells with positive staining for E‐cadherin, ZO‐1, MMP‐9, MMP‐2, TIMP‐2 or TIMP‐3 were counted in 10 high‐power fields and expressed as means ± SD. Data are expressed as the mean ± SD and analyzed using one‐way ANOVA with Tukey's post‐hoc test. All data were obtained from three independent experiments. *P < 0.05, **P < 0.01, ***P < 0.001. Scale bar = 100 μm. AMI‐1, arginine methyltransferase inhibitor 1; CLP, cecal ligation and perforation; PRMT1, EMT, epithelial–mesenchymal transition; protein arginine methyltransferase‐1; SI‐AKI, sepsis‐induced acute kidney injury.

To verify the regulatory role of PRMT1 on the connexins and matrix proteinases through TGF‐β1 signal pathway in SI‐AKI, we performed immunofluorescence to quantify and localize the connexin and the matrix proteinases using mice renal tissue sections. The protein expression of E‐cadherin and ZO‐1 was stable in the mice from the sham and sham with AMI‐1 groups (Fig. 3A). However, these proteins were subtly detected in the kidneys of the CLP‐operated mice group. After AMI‐1 pretreatment, the expression levels of E‐cadherin and ZO‐1 were partially restored (*P* < 0.01). As shown in Fig. 3B,C, the expression levels of matrix metalloproteinase (MMP)‐9 and MMP‐2 were significantly increased in the CLP‐treated mice groups compared to those in the sham‐operated mice group (*P* < 0.01), whereas the expression of tissue inhibitor of metalloproteinase (TIMP)‐2 and TIMP‐3 proteins, which were expressed in normal renal tubules presented an opposite trend (*P* < 0.01). AMI‐1 treatment decreased the levels of MMP2 and MMP9 and partially restored those of TIMP‐2 and TIMP‐3 in the renal tissue. These results reveal that PRMT1 inhibition ameliorates CLP‐induced loss of intercellular adherent junctions, as well as matrix metalloproteinase secretion in the kidney.

**Fig. 4:**
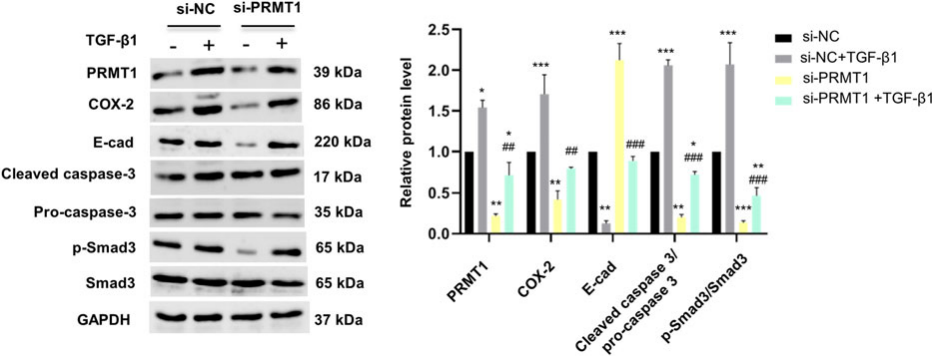
Knockdown of PRMT1 inhibits TGF‐β1 induced inflammation‐, apoptosis‐, and EMT‐related protein expression. Western blot assay images and the relative protein levels of PRMT1, COX‐2, E‐cadherin, cleaved caspase‐3, pro‐caspase‐3, and p‐Smad3 in si‐PRMT‐transfected mouse renal tubular epithelial cells (mRTECs) with or without TGF‐β1. Data are expressed as the mean ± SD and analyzed using one‐way ANOVA with Tukey's post‐hoc test. All data were obtained from three independent experiments. *P < 0.05, **P < 0.01, and ***P < 0.001 vs. si‐NC; ^##^ *P* < 0.01, and ^###^ *P* < 0.001 vs. si‐NC + TGF‐β1. EMT; epithelial–mesenchymal transition; PRMT1, protein arginine methyltransferase‐1.

To verify the biological role of PRMT1 regulating SI‐AKI in mice through TGF‐β1 signal pathway, we performed experiments on PRMT1 regulating the association molecules of TGF‐β1 signal pathway in mouse renal tubular epithelial cells (mRTECs). The expression levels of PRMT1, E‐cadherin, cleaved caspase‐3, pro‐caspase‐3, phosphorylated Smad3 (p‐Smad3), and total Smad3 were examined in mRTECs stimulated by TGF‐β1 or normal saline along with siRNA targeting PRMT1 (si‐PRMT1) or siRNA control (si‐NC) (Fig. 4). Under TGF‐β1 stimulation, the expression of PRMT1, COX‐2, cleaved caspase‐3, and p‐Smad3 in si‐NC‐transfected cells was significantly increased (*P* < 0.05). However, E‐cadherin expression was decreased (*P* < 0.01). There was no significant difference in the total protein expression of pro‐caspase‐3 and Smad3 among the groups. Meanwhile, PRMT1 knockdown mediated by small interfering RNA (siRNA) downregulated the expression of COX‐2, cleaved caspase‐3, and p‐Smad3 (*P* < 0.01) but upregulated the expression of E‐cadherin (*P* < 0.001) in the presence of TGF‐β1, suggesting that PRMT1 knockdown may reduce TGF‐β1‐induced epithelial cell inflammation, EMT, and apoptosis *in vitro*.

**Fig. 5:**
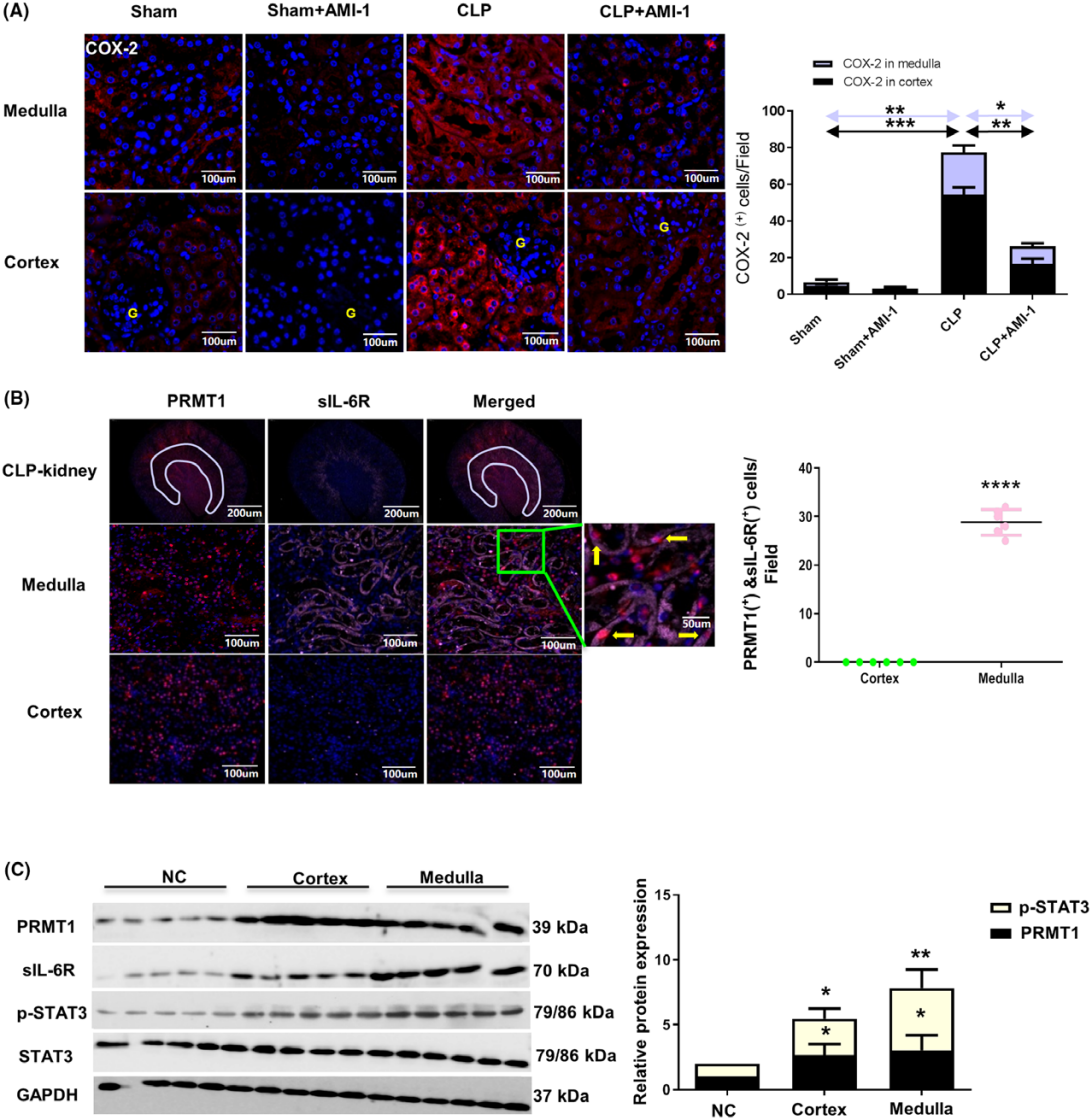
The inflammatory marker COX‐2 is mainly expressed and IL‐6 trans‐signaling is activated in the medulla. (A) Immunofluorescence staining of COX‐2 and quantitative analysis of COX‐2^(+)^ cells in the renal medulla and cortex. The yellow ‘G’ indicates glomerular. Scale bar = 100 μm. (B) Immunofluorescence staining of sIL‐6R and quantitative analysis of sIL‐6R^(+)^ and PRMT1^(+)^ cells in medulla, cortex or a whole kidney of CLP‐operated mice. Scale bar = 50 μm and 100 μm. (C) Western blot assay of PRMT1, sIL‐6R, pSTAT3, and total STAT3 in the cortex and medulla and the relative expression levels of PRMT1 and p‐STAT3 in the cortex and medulla. Data are expressed as the mean ± SD and analyzed using one‐way ANOVA with Tukey's post‐hoc test. All data were obtained from three independent experiments. *P < 0.05, **P < 0.01, ***P < 0.001 and ****P < 0.0001 vs. NC groups. CLP, cecal ligation and perforation; PRMT1, protein arginine methyltransferase‐1; sIL‐6R, soluble IL‐6R.

Whether PRMT1 contributes to renal inflammation need to be investigated because renal tissue is exposed to severe inflammatory cytokine storm during sepsis, and inflammation is an important mechanism for fibrosis [30]. Therefore, we assessed the expression of COX‐2 protein, which is an important inflammatory marker [31]. COX‐2 protein was increased in the cortex and medulla of mice in CLP‐treated group, and reduced in that of mice in the CLP + AMI‐1 group (*P* < 0.01) (Fig. 5A). However, the mechanism of this expression cannot be explained by TGF‐β1 alone, which was predominantly observed in the cortex. We speculated that it might be related to sepsis toxin, and thus we investigated sIL‐6R, which is the receptor of the IL‐6 trans‐signaling pathway. In immunofluorescence, PRMT1 (red) was evenly distributed in the cortex and medulla, whereas sIL‐6R (pink) protein was concentrated mainly in the medulla (Fig. 5B). Next, we isolated the renal cortex and medulla from CLP‐treated mice and extracted all proteins from the kidneys. We found that there was minimal expression of PRMT1, sIL‐6R, and phosphorylated STAT3 (p‐STAT3) in the normal renal tissue, whereas elevated expression levels of these proteins occurred in the cortex and medulla of CLP treated‐mice (Fig. 5C). The expression levels of PRMT1 and p‐STAT3 in the medulla were significantly higher than those in the cortex. STAT3 is a key downstream molecule of the sIL‐6R receptor mediated IL‐6 trans‐signal pathway, and an increase in the expression of p‐STAT3 reveals the activation of the inflammatory factor IL‐6 trans‐signal pathway [20, 25]. These results show that PRMT1 is related to the IL‐6 trans‐signal pathway, namely the inflammatory pathway.

**Fig. 6:**
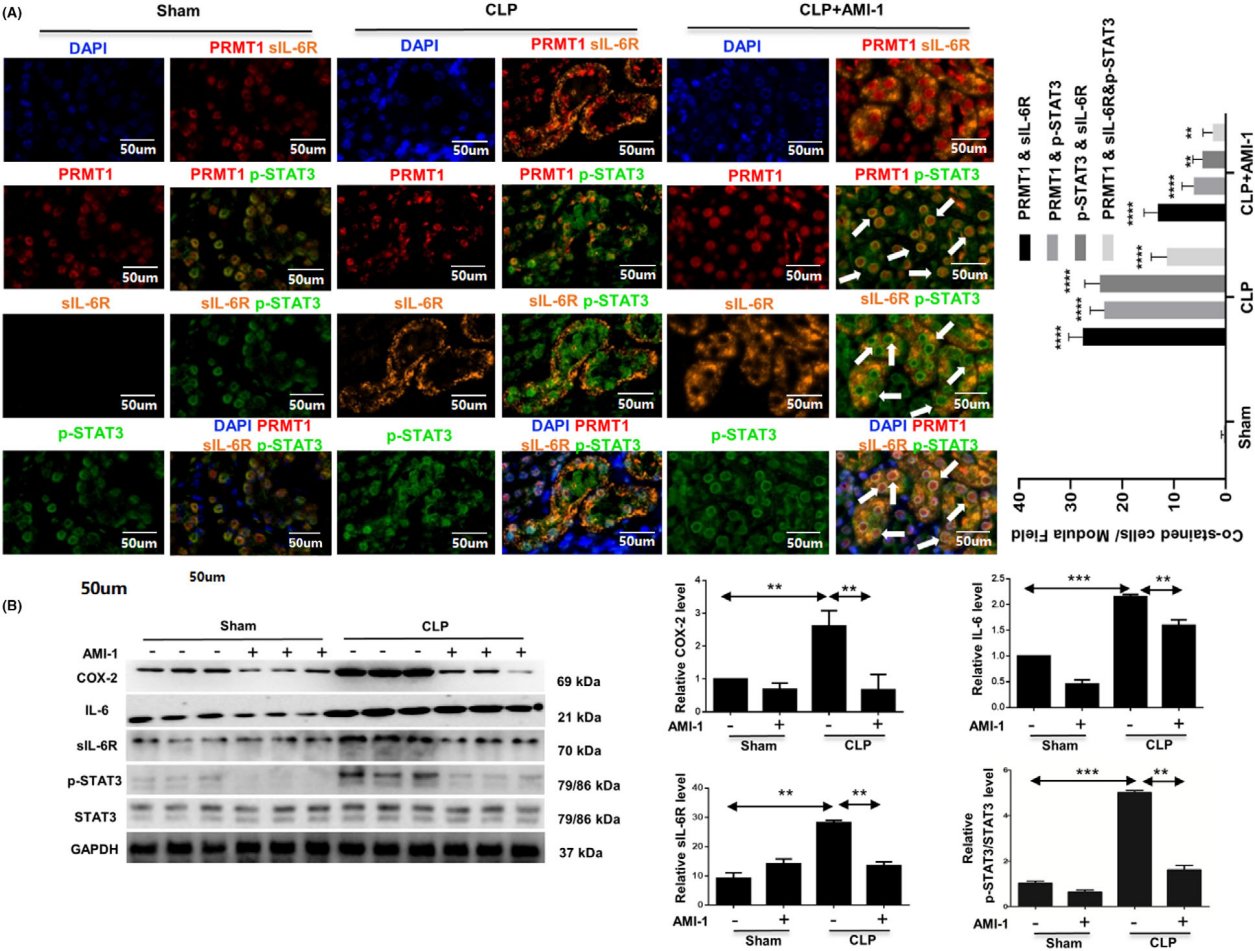
The expression of PRMT1, sIL‐6R, p‐STAT3, COX‐2, IL‐6 and STAT3 in the kidneys. (A) Immunofluorescence staining of PRMT1, sIL‐6R, p‐STAT3, and their co‐localization in the kidneys. The number of PRMT1^(+)^ and sIL‐6R^(+)^ cells, PRMT1^(+)^ and pSTAT3^(+)^ cells, p‐STAT3^(+)^ and sIL‐6R^(+)^ cells, and PRMT1^(+)^ and sIL‐6R^(+)^ & pSTAT3^(+)^ cells. Tubular cells with positive staining for these proteins were counted in 10 high‐power fields and expressed as the mean ± SD. (B) Western blot assay of COX‐2, IL‐6, sIL‐6R, p‐STAT3, and total STAT3 and their relative levels in mouse kidneys. Data are expressed as the mean ± SD and analyzed using one‐way ANOVA with Tukey's post‐hoc test. All data were obtained from three independent experiments. **P < 0.01, ***P < 0.001 and ****P < 0.0001 vs. Sham. Scale bar = 50 μm and 100 μm. PRMT1, protein arginine methyltransferase‐1; sIL‐6R, soluble IL‐6R.

Immunofluorescence staining was used to analyze the expression of PRMT1, sIL‐6R, and p‐STAT3 in renal tubular epithelial cells of different groups of mice. In sham‐operated kidneys, PRMT1 was occasionally detected in the nuclei of the renal tubular epithelial cells, and p‐STAT3 was mainly expressed in the cytoplasm of the tubular epithelial cells, whereas sIL‐6R could scarcely be found (Fig. 6A). In the renal tissues from CLP‐treated mice, PRMT1 was highly expressed in the nuclei of renal tubular epithelial cells, and p‐STAT3 appeared to be mostly transferred into the cells. After AMI‐1 treatment, the number of PRMT1 and sIL‐6R co‐expressing cells decreased significantly (*P* < 0.001). Interestingly, p‐STAT3, originally detected in the nuclei, appeared to translocate to the cytoplasm. Subsequently, using western blot analysis, we further analyzed whether inhibition of PRMT1 can block the IL‐6/sIL‐6R/STAT3 signaling pathway to prevent renal inflammation. Compared with the mice from the sham group, the expression levels of COX‐2, IL‐6, sIL‐6R, and p‐STAT3 were significantly increased after CLP operation, whereas AMI‐1 injection downregulated the expression levels of these proteins in both the sham and CLP groups (*P* < 0.01), suggesting that inhibition of PRMT1 stopped the inflammation induced by sepsis by blocking activation of STAT3 (Fig. 6B).

**Fig. 7:**
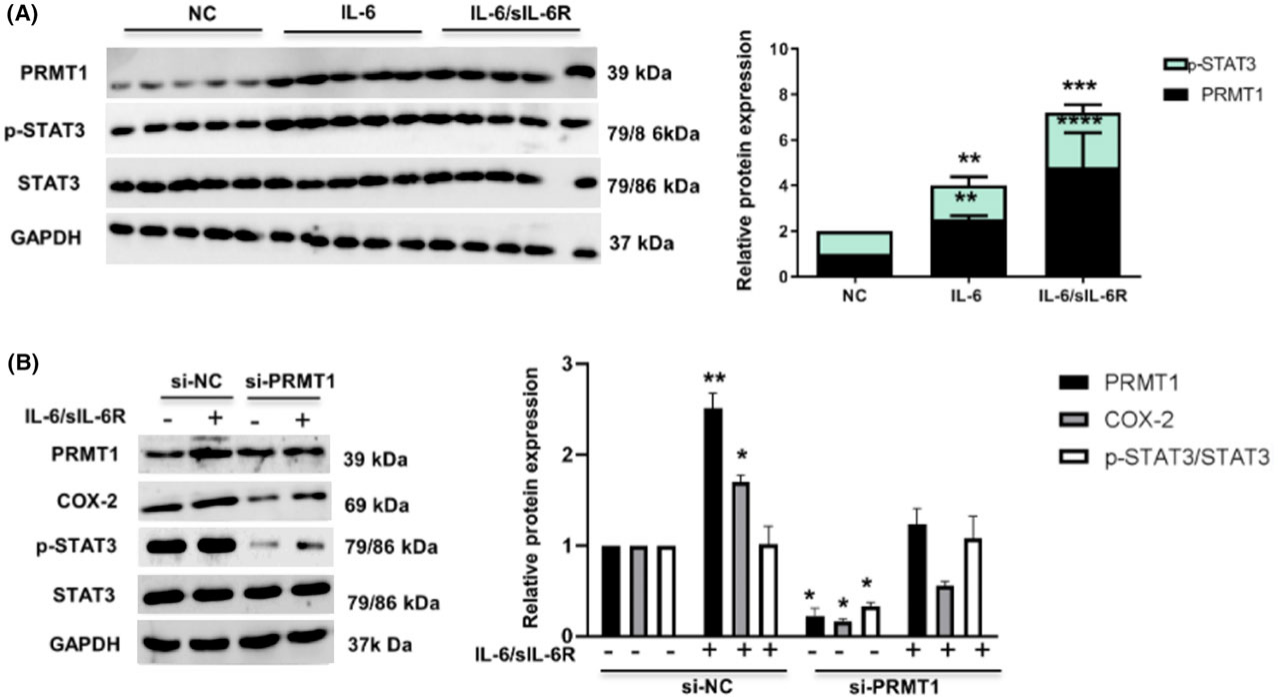
Knockdown of PRMT1 represses the activation of IL‐6 trans‐signaling. (A) Western blot detection of PRMT1, p‐STAT3, and total STAT3 in mRTECs treated with IL‐6 or IL‐6 and sIL‐6R complex and the relative expression levels of p‐STAT3 and PRMT1. **P < 0.01, ***P < 0.001, and ****P < 0.0001 vs. NC. (B) Western blot assay of PRMT1, COX‐2, p‐STAT3, and total STAT3 and their relative expression in si‐PRMT1‐transfected mRTECs under L‐6 and sIL‐6R complex treatment. Data are expressed as the mean ± SD and analyzed using one‐way ANOVA with Tukey's post‐hoc test. All data were obtained from three independent experiments. *P < 0.05 and **P < 0.01 vs. si‐NC without IL‐6/sIL‐6R. PRMT1, protein arginine methyltransferase‐1; sIL‐6R, soluble IL‐6R.

Thus, to verify the biological role of PRMT1 regulating SI‐AKI in mice through IL‐6 trans‐signal pathway, we performed experiments on PRMT1 regulating the association molecules of the IL‐6 trans‐signal pathway in mRTECs. As shown in Fig. 7A, the expression levels of PRMT1, p‐STAT3, and total STAT3 were detected in renal tubular epithelial cells. After 12 h of IL‐6 stimulation, the expression levels of PRMT1 and p‐STAT3 slightly increased (*P* < 0.01), and the total expression levels of STAT3 did not change significantly. When supplemented with both IL‐6 and sIL‐6R, the expression levels of PRMT1 and p‐STAT3 increased significantly (*P* < 0.001). These results indicate that IL‐6 and sIL‐6R complex upregulate the expression of PRMT1 and p‐STAT3 phosphorylation, as well as activate the IL‐6 trans‐signal pathway. When siRNA was used to knock down the PRMT1 expression, we observed changes in the expression levels of COX‐2, p‐STAT3, and total STAT3 after stimulation with IL‐6 and sIL‐6R complex (Fig. 7B). The expression levels of these proteins in si‐PRMT1‐transfected cells were significantly lower than those in the si‐NC‐transfected cells without stimulation (*P* < 0.05). The stimulation of IL‐6 and sIL‐6R complexes significantly increased the expression of PRMT1, COX‐2, and p‐STAT3 in si‐PRMT1‐transfected cells. There was no significant difference in the total expression of total STAT3 protein among these groups (*P* > 0.05). The results reveal that PRMT1 molecule was associated with the IL‐6 trans‐signal pathway, mediating biological effects.

## Discussion

Renal ischemia–reperfusion induced by septic shock is the main factor contributing to AKI. The underlying pathway by which PRMT1 regulates the progression of SI‐AKI has been rarely reported. In the present study, SI‐AKI model was induced through CLP operation, and overexpression of PRMT1 was observed in the model. *In vivo*, inhibition of PRMT1 by AMI‐1, an inhibitor of PRMT1, alleviated kidney damage by repressing cell apoptosis, inflammation, and EMT through both the TGF‐β1 signaling pathway in the cortex and IL‐6 trans‐signaling pathway in medulla. *In vitro*, these results were further verified with siRNA‐mediated knockdown of PRMT1 in mRTECs.

The PRMT1 molecule is associated with many biological functions of the cell through different signaling pathways, and may even play contradictory roles in different stimulators [11, 12]. A previous study found that PRMT1 is associated with streptozocin‐induced pancreatic β‐cell injury, and its downregulation mediated by microRNA or circRNA improves insulin secretion [32]. Under normal conditions, PRMT1 restricts hepatocyte proliferation [33]. However, a recent study indicates that PRMT1 protects the liver from alcohol‐induced injury by regulating oxidative stress responses [34]. Some studies indicate that PRMT1 is upregulated in angiotensin II‐induced renal fibrosis [11, 35]. Therefore, clarifying the biological role of PRMT1 in different diseases is helpful in clinical treatment, especially for common refractory diseases. In the present study, sepsis induced PRMT1 expression in the renal, and inhibition of PRMT1 decreased the serum level of Cr and BUN and the expression of NGAL, a marker of renal injury, demonstrating that PRMT1 contributes to SI‐AKI. However, the role of PRMT1 in SI‐AKI remains unclear.

EMT, a vital process in kidney injury [4, 36, 37], is characterized by two major pathological attributes; namely, the loss of tight junction proteins of tubules (e.g. E‐cadherin, ZO‐1) [38, 39] and damage of tubular basement membrane mediated by MMP secretion or TIMP degradation [40, 41, 42, 43]. We found that sepsis enhanced the expression of E‐cadherin, ZO‐1, MMP2, and MMP9 but weakened that of TIMP‐2 and TIMP‐3. Additionally, the inhibition of PRMT1 was demonstrated to reverse these EMT‐related proteins and suppresses sepsis‐induced apoptosis in renal tissues. Notably, PRMT1 is associated with both EMT and apoptosis pathological processes in SI‐AKI. Another study reports that PRMT1 induces ER stress and epithelial‐mesenchymal transition in renal tubular epithelial cells and apoptosis, thereby facilitating diabetic nephropathy [44]. According to studies, obesity‐related insulin resistance will lead to an increase in cytokine IL‐6, which itself will lead to the occurrence of inflammation and increase albuminuria [45], and so inhibition of cytokines and thus inflammation may be a therapeutic target to improve and prevent diabetic nephropathy [46]. TGF‐β1 can regulate the disorder of EMT through phosphorylation of Smad2 and Smad3 by activating transmembrane serine/threonine kinases [47, 48]. Our results showed that, in the SI‐AKI murine model, TGF‐β1/Smad3 signaling was activated upon sepsis‐induced kidney injury, resulting in increased expression levels of MMPs and TIMPs. As a result of the abundant blood supply in the renal cortex, TGF‐β1 detected in the renal cortex likely originates from the blood system rather than the collecting duct system. Previous studies have shown that PRMT1 regulated the activation of renal tubulointerstitial fibroblasts through the TGF‐β1/Smad3 signaling pathway [16]. Additionally, PRMT1 is found to promote EMT through the TGF‐β1/Smad pathway in hepatic carcinoma cells [49], and is required for TGF‐β‐induced Smad3 activation, as well as promoting TGF‐β‐induced EMT [50]. These data show that PRMT1 regulates EMT through the TGF‐β1/Smad pathway. Our results show that, during acute renal injury, PRMT1 mediates the trans‐differentiation, inflammatory injury, and apoptosis of renal tubular epithelial cells via TGF‐β1. Moreover, in SI‐AKI, these processes predominantly occurred in the renal cortex and part of the medullary outer zone, comprising the proximal and distal distribution of renal tubules, rather than the collecting duct system.

**Fig. 8:**
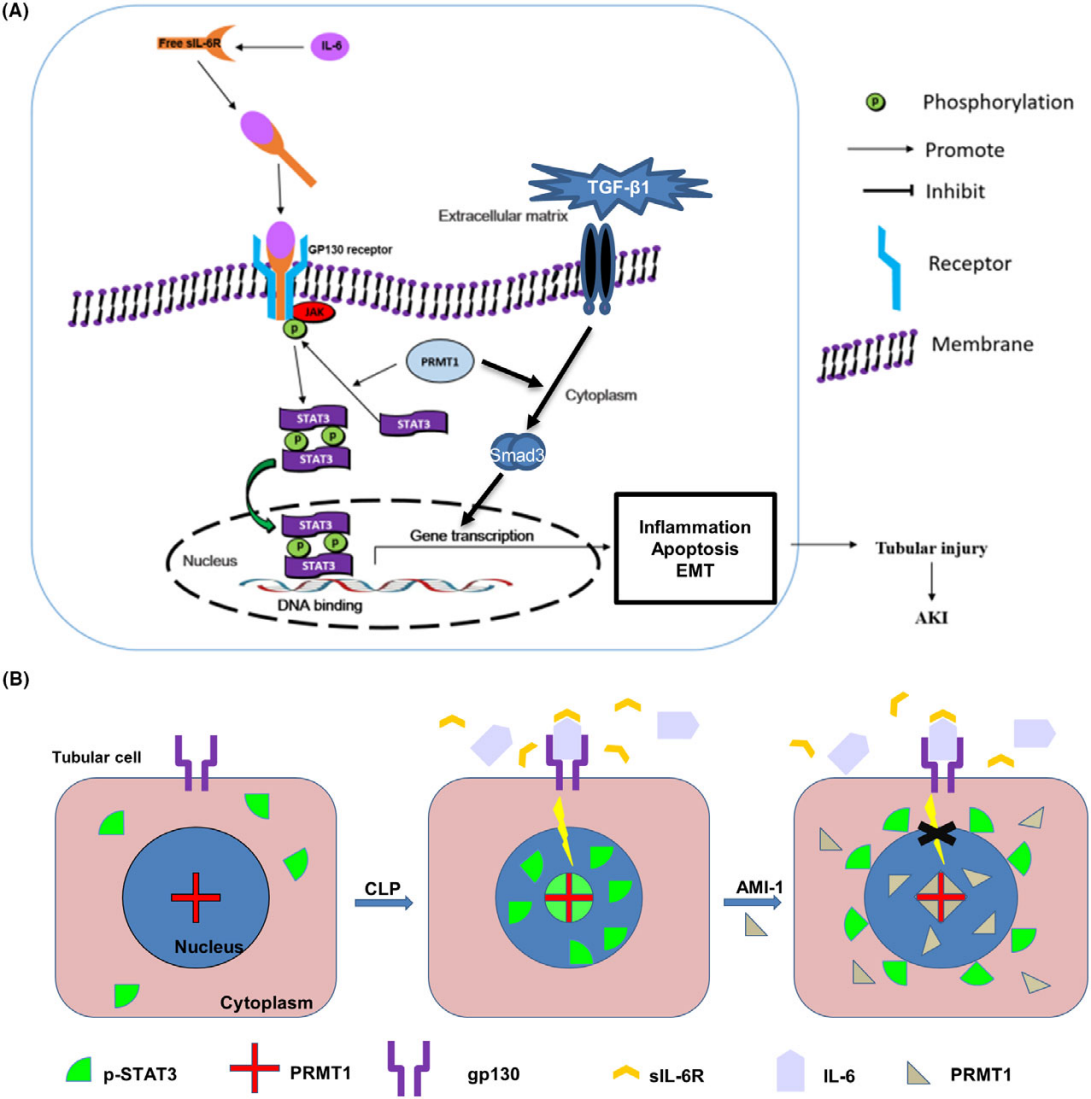
Diagram of the regulatory mechanism of RPMT1 in SI‐AKI. (A) Schematic cartoon of how PRMT1 regulates the TGF‐β1/Smad3 and IL‐6 trans‐signaling pathways. (B) Schematic cartoon of how AMI‐1 blocks IL‐6/sIL‐6R induced STAT3 activation. AMI‐1, arginine methyltransferase inhibitor 1; CLP, cecal ligation and perforation; PRMT1, protein arginine methyltransferase‐1; sIL‐6R, soluble IL‐6R.

Surprisingly, COX‐2 was primarily detected in the renal tubular epithelial cells in the medullary zone and cortex with even distribution, which is significantly different from that of TGF‐β1 in the tubulointerstitium. The results indicate that the inflammatory response in medullary region may not be mediated by TGF‐β1, and another main signaling pathway may possibly regulate the inflammation in medulla. The IL‐6 mediating pathway includes both IL‐6 classic and trans‐signaling pathways [22] and phosphorylation of STAT3 is a common outcome of the two pathways. IL‐6 trans‐signaling via the sIL‐6R is somewhat pro‐inflammatory [51]. Blockade of IL‐6 trans‐signaling prevents renal fibrosis by suppressing STAT3 activation [26]. Knockdown of PRMT1 is demonstrated to inhibit inflammatory responses in human chondrocytes [52]. However, the role of PRMT1 in the IL‐6 trans‐signaling pathway remains unelucidated. Our results showed that inhibition of PRMT1 downregulates the expression of COX‐2, IL‐6, sIL‐6, and p‐STAT3 in the kidney, which was induced by sepsis, revealing the regulation role of PRMT1 in inflammation and the IL‐6 trans‐signaling pathway. The expression levels of sIL‐6R and p‐STAT3 in the medulla were significantly higher than those in the cortex, indicating that the regulation of inflammation may be mainly in the medulla and regulated by the IL‐6‐mediated signaling pathway. The expression of p‐STAT3 in the sepsis‐induced injured kidney was significantly higher than that in the sham‐operated kidney. Furthermore, IL‐6 alone or IL‐6/sIL‐6 complex can increase the expression level of PRMT1 in cultured tubule epithelial cells, and the significant increase in p‐STAT3 expression was mediated by IL‐6/sIL‐6 complex. The regulatory mechanism of PRMT1 was identified in the IL‐6 trans‐signaling pathway, and si‐PRMT1‐transfected cells further confirmed such a regulatory effect. In the sham operation group, a moderate amount of p‐STAT3 expression was detected in the renal medulla, and there was minimal to no expression of sIL‐6R or co‐localization with PRMT1. However, using immunofluorescence detection, the p‐STAT3 in the AMI‐1 treated groups resulted in obstruction inside the nucleus via PRMT1 inhibition. Therefore, we hypothesized that only methylated STAT3 could be phosphorylated or only after the methylation by PRMT1 that activated STAT3 can translocate to the nucleus, subsequently inducing signals. PRMT1 may enhance STAT3 phosphorylation and helps p‐STAT3 protein enter the nucleus to initiate gene expression, which contributes to EMT, inflammation, and apoptosis in SI‐AKI (Fig. 8A). Treatment by AMI‐1 or siRNA weakened the methylated activity of PRMT1 in septic kidney cells; thus, non‐methylated STAT3 could not alter the conformation to pass through the nuclear pore (Fig. 8B). Thus, identification of the process by which p‐STAT3 is able to enter the nucleus with or without PRMT1 methylation in septic renal cells is warranted.

The present study did not further investigate the correlation between TGF‐β/Smad3 and the IL6/STST3 pathway, but according to a study by O'Reilly *et al*. [27], the TGF‐b pathway appears to be related to the STAT3 pathway. IL‐6 trans‐signaling drives the STAT3‐dependent pathway, leading to overactive TGF‐β promoting Smad3 activation and fibrosis via Gremlin protein. Therefore, the relationship between TGF‐β and STAT3 remains unclear and requires further investigation.

## Conclusions

We have established a new understanding of the mechanism of epigenetic regulation of septic kidney injury. We found that PRMT1 regulates renal tubular injury through the TGF‐β1 signaling pathway in the cortex and the IL‐6 trans‐signaling pathway in the medulla.

## Materials and methods

### Cell culture and treatment

The mRTECs were obtained from American Type Culture Collection (ATCC, Manassas, VA, USA). The cells were grown in F12 medium supplemented with 10% fetal bovine serum and incubated at 37 °C in 5% CO_2_ atmosphere as previously described [16]. To knock down PRMT1 in mRTECs, the transfection of siRNA targeting PRMT1 (si‐PRMT1) or its siRNA control (si‐NC) into mRTECs was performed using Lipofectamine 2000 transfection reagent (RiboBio, Guangzhou, China) in accordance with the manufacturer's instructions. All cells were cultivated for 24 h after transfection. When needed, the transfected cells were incubated with the culture medium containing TGF‐β1 (2 ng·mL^−1^; Abcam, Cambridge, MA, USA), IL‐6 (50 ng·mL^−1^; Abcam) or sIL‐6R (200 ng·mL^−1^; Abcam). An equal amount of normal saline served as negative control for cell treatment.

### Animals and surgery

All animal experiments were conducted strictly following the international ethical guidelines and the National Institutes of Health Guide concerning the Care and Use of Laboratory Animals. The experimental protocols were approved by the Institutional Animal Care and Use Committee of Anhui Provincial Hospital of China (Approval No.: 2019 KY LSN No. 41). Twenty‐four Germ‐free C57BL male mice (weight 20 ± 0.98 g) were purchased from the Model Animal Research Center of Nanjing University (MARC, Nanjing, China). All mice were randomly divided into four experimental groups (*n* = 6 per group): sham operation, sham operation with AMI‐1 (a PRMT1 inhibitor; Sigma‐Aldrich, St Louis, MO, USA) treatment, CLP, and CLP with AMI‐1 treatment. The animals were stored humanely at 23 °C under a 12 : 12 h light/dark photocycle with free access to food and water to alleviate suffering. According to our own research protocol, the mice were fasted for 12 h but had access to water before the experiment.

For SI‐AKI model surgical procedures, the mice were anesthetized with pentobarbital sodium at a dose of sodium pentobarbital (50 mg·kg^−1^) intraperitoneally. Based on previous study [53], CLP operation was performed on 12 mice to induce sepsis. Briefly, under aseptic conditions, a laparotomy was conducted through a 2‐cm ventral midline abdominal incision to allow exposure of the cecum with adjoining intestine. Subsequently, the bottom of the cecum below the ileocecal valve was tightly ligated with 3.0 silk suture and punctured twice with a no. 18 needle. The cecum was gently squeeze and small amount of feces was squeezed out from the perforated part. The cecum was returned to the abdominal cavity, and the laparotomy incision was closed with 4–0 polylactic acid suture. After the operation, 5 mL·100 g^−1^ warm (37 °C) isotonic saline was injected intraperitoneally to supplement blood loss; the mice were then placed in cages. The sham‐operated mice underwent laparotomy through a midline incision without the cecum puncture. Mice in the sham with AMI‐1 and CLP with AMI‐1 groups were injected intraperitoneally with 10 mg·kg^−1^ AMI‐1 diluted in DMSO before sham or CLP operation. The sham operation and CLP groups were injected with an equal dose of DMSO to serve as controls. After 18 h post‐surgery, all mice were killed, and peripheral blood and kidney tissues were collected for subsequent assays.

### Measurements of blood biochemistries

Blood samples were collected from the tail vein of all mice 18 h after CLP operation for measuring serum Cr and BUNlevels. Then blood was centrifuged at 3000 r.p.m. for 5 min. The serum was collected and assessed using the Cr and BUN kit (Mindray Medical Corp, Shenzhen, China) in accordance with the manufacturer's instructions.

### Western blot assay

Western blot assay was performed as previously described [16]. Briefly, proteins isolated from the kidneys of mice were quantified using a BCA kit (Beyotime, Shanghai, China). The proteins were then separated by SDS/PAGE and transferred onto poly(vinylidene difluoride) membranes (Millipore, Billerica, MA, USA), which were subsequently incubated overnight at 4 °C with the primary antibodies (dilution 1 : 1000). Antibodies against NGAL (ab216462), PRMT1 (ab73246), GAPDH (ab9483), Smad3 (ab84177), p‐Smad3 (ab52903), E‐cadherin (E‐cad) (ab231303), pro caspase‐3 (ab32499), COX‐2 (ab179800), p‐STAT3 (ab76315), and STAT3 (ab68153) were purchased from Abcam (Cambridge, UK). Antibody against sIL‐6R (23457) was purchased from Proteintech (Rosemont, IL, USA). Antibody against cleaved caspase‐3 (SAB4503292) was purchased from Sigma‐Aldrich. The membranes were then incubated with the secondary antibody coupled with horseradish peroxidase at room temperature for 30 min. The bands of proteins were visualized via enhanced chemiluminescence substrate (Solarbio, Beijing, China). GAPDH served as an internal control.

### Immunofluorescence staining

Immunofluorescence staining was performed following a protocol published previously [16]. Briefly, paraffin‐embedded kidney slices underwent deparaffinization, hydration, and antigen retrieval. Subsequently, the sections were incubated with 1% BSA (Aladdin, Shanghai, China) in phosphate‐buffered saline‐Tween 20 for 1 h followed by three washes in 1 × phosphate‐buffered saline‐Tween 20. The tissue slides were probed with the primary antibody against E‐cadherin (E‐cad) (ab231303), ZO‐1 (ab221547), MMP‐2 (ab92536), MMP‐9 (ab76003), TIMP‐2 (ab230511), TIMP‐3 (ab39184), RMPT1, COX‐2, p‐STAT3 or sIL‐6R at 4 °C overnight and fluorescence‐labeled secondary antibodies (A0562; Beyotime) diluted in 1% BSA at 25 ± 5 °C for 1 h. The coverslips were imaged under microscopy after co‐staining with 4′,6‐diamidino‐2‐phenylindole (Aladdin). For immunofluorescence double staining, the tissue slices were incubated with a mixture of different primary antibodies against proteins of interest overnight at 4 °C.

### Immunohistochemistry

The protocol for immunohistochemistry was performed as previously described [16]. Briefly, the tissue sections on the slides were deparaffinized and rehydrated. The endogenous peroxidase was inactivated using 3% H_2_O_2_. Pre‐incubation of 1.5% BSA was performed before incubation with primary antibody to reduce background staining. Primary antibodies against TGF‐β1 (SAB4502954; Sigma‐Aldrich) and cleaved caspase‐3 (SAB4503292; Sigma‐Aldrich) and secondary antibodies were diluted in 1% BSA and used to incubate the slides sequentially. Subsequently, the sections were stained with 3,3′‐diaminobenzidine (Sigma‐Aldrich) substrate and counterstained with hematoxylin. The slides were then developed and imaged(CX23LEDRFS1C; Olympus, Tokyo, Japan).

### TUNEL staining

Apoptosis in the renal tissues was detected using the *In Situ* Cell Death Detection Kit (Roche, Mannheim, Germany). Deparaffinized and rehydrated kidney sections were incubated with TUNEL reaction mix at 37 °C for 60 min in the dark. The nucleus was labeled with 4′,6‐diamidino‐2‐phenylindole, and TUNEL‐positive cells were evaluated using fluorescence microscopy.

### Statistical analysis

Data are expressed as the mean ± SD. Statistical significance was determined using Student's *t*‐test or one‐way analysis of variance (ANOVA). *P* < 0.05 was considered statistically significant. Statistical analysis was performed using prism, version 7 (GraphPad Software Inc., San Diego, CA, USA).

## Conflicts of interest

The authors declare that they have no conflicts of interest.

## Author contributions

YZ and WL contributed to the study conceptualization. YZ, LW, RL, XD, SY and YC contributed to investigations. YZ, LW, RL, and CZ contributed to the formal analysis. YZ, LW, ZW and WL contributed to writing the original draft. YZ, LW, RL, XD, and WL contributed to funding acquisition.

## Data Availability

The data used in this study are available from the corresponding authors upon reasonable request.
